# Side Chain Crosslinked Anion Exchange Membrane for Acid Concentration by Electrodialysis

**DOI:** 10.1021/cbe.4c00096

**Published:** 2024-07-10

**Authors:** Haoyang He, Qian Chen, Rongqiang Fu, Zhaoming Liu, Liang Ge, Tongwen Xu

**Affiliations:** † Key Laboratory of Precision and Intelligent Chemistry, School of Chemistry and Materials Science, University of Science and Technology of China, Hefei, 230026, China; ‡ Applied Engineering Technology Research Center for Functional Membranes, Institute of Advanced Technology, University of Science and Technology of China, Hefei, 230088, China; § Shandong Tianwei Membrane Technology Co., LTD, Weifang, 262737, China

**Keywords:** Anion exchange membrane, In situ crosslink, Acid-block, Electrodialysis, Hydrogen ions

## Abstract

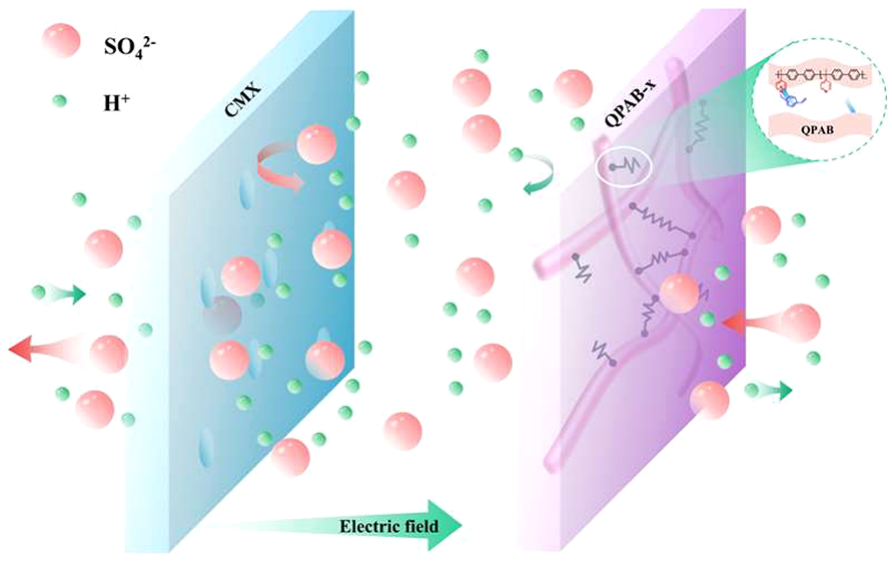

Electrodialysis (ED) technology for waste acid treatment has high economic efficiency and environmentally friendly advantages. The primary limitation of ED in the retrieval of low-concentration spent acids lies in the leakage of hydrogen ions through anion exchange membranes (AEMs) due to its extremely small size and high mobility. To address this issue, a series of AEMs named QPAB-*x* (*x* = 3, 5, 7, 10) were designed for acid concentration in ED process by increasing the membrane densities through in situ crosslinking in this study. The successful synthesis of polymers was confirmed through ^1^H nuclear magnetic resonance hydrogen (^1^H NMR) spectroscopy and Attenuated total reflection-Fourier transform infrared (ATR-FTIR) spectroscopy. Furthermore, ATR-FTIR spectroscopy showed that the higher the side chain content, the higher the crosslinking degree of the membranes. X-ray photoelectron spectroscopy (XPS) was employed to characterize the effects of aqueous and acidic environments on QPAB membranes. The performance disparities between QPAB-*x* membranes in acidic and aqueous environments were examined separately. Subsequently, the influence of crosslinking degree on the acid-blocking capability of the membranes was thoroughly investigated by conducting ED acid-concentration experiments to monitor the hydrogen ions concentration process and determine the current efficiency and energy consumption of the QPAB-*x* membranes. Our experimental results demonstrated that QPAB-*x* membranes with higher cross-linking degrees have lower water content, especially the QPAB-10 membrane with an IEC of approximately 1.5 mmol g^–1^ and a remarkably low water content of around 10%. This leads to a reduced H^+^ transfer number and excellent acid-blocking properties. Additionally, compared to commercial membrane A2, using the QPAB-10 membrane in the ED process resulted in a higher final H^+^ concentration in the concentrated chamber. Consequently, these synthesized membranes exhibit considerable promise in the field of ED acid recovery.

## Introduction

Sulfuric acid is widely used in various industries, such as chemical, metallurgical, and petroleum sector. The increasing demand for sulfuric acid has led to a rise in waste acid generation from industrial activities. However, directly utilizing this waste acid is challenging due to its low concentrations and requires complex treatment processes.^1^ Discharging the waste acid into the environment not only causes pollution but also wastes valuable resources. Consequently, the recycling of waste acid has gained increasing attention due to the progressively stringent environmental regulations.^2,3^

Membrane technology, as a nascent treatment technology, offers economic efficiency and environmental friendliness.^4^ Specifically, diffusion dialysis (DD) and electrodialysis (ED) technologies are used for effective separation and recovery of acid components in acidic waste liquid. DD is effective for treating high-concentration waste acid, while ED offers advantages in treating low-concentration waste acid from an economic perspective.^5−8^ ED utilizes ion exchange membrane’s selective permeability to separate anions and cations, concentrate and desalinate substances.^9^ However, the issue of acid leakage poses a challenge in ED technology.^10^ Traditional anion exchange membranes (AEMs) exhibit permeability to H^+^ ions due to their distinct attributes like higher migration rate compared to other ions, smaller size of H^+^ ions themselves along with quantum-mechanical “tunnel effect” facilitating rapid transfer within water molecules.^11^ Consequently, these AEMs face limitations regarding acid recovery efficiency and concentration which impact current efficiency and energy consumption levels.^10^ Thus, the development of acid-blocking AEMs with exceptional performance is crucial for acid concentration and recovery through ED technology.^12−14^

Previous research has highlighted the role of water molecules in facilitating the transportation of H^+^ ions via the Grotthuss mechanism and/or the vehicle mechanism.^15−17^ Both mechanisms rely on water molecules for the movement of H^+^ ions. Reducing the water content of AEMs is expected to impede transmembrane transportation of H^+^ ions, consequently leading to an acid blocking membrane. To achieve acid blocking, different approaches have been explored. One strategy is enhancing membrane density by developing a densely cross-linked layer on its surface, effectively blocking acid penetration.^18^ Another proposed solution involves decreasing water uptake and introducing hydrophobic groups into the membrane through incorporating hydrophobic groups, increasing crosslinking degree, and utilizing weak base functional groups.^19,20^ For example, researchers successfully developed acid-blocking membranes by polymerizing functional monomers containing pyrrole groups on the AEM surface, resulting in a compact thin layer that improves their ability to block acid penetration.^21^ Another study successfully synthesized AEMs with a semi-interpenetrating polymer network structure by polymerizing and crosslinking hydrophobic poly(vinyl chloride), dimethylammonioethyl methacrylate containing a weak base tertiary amine and divinylbenzene. The resulting membranes were further treated with iodomethane to enhance their acid-blocking properties.^22^ Incorporating graphene oxide into a polymer increased water uptake of membranes, intensifying the migration of H^+^.^23^ Crosslinking with polychloromethylstyrene enhanced membrane compactness and successfully reduced the water uptake.^24^ Surface-initiated crosslinking of AEMs with α,α′-dibromo-*p*-xylene constructed crosslinked AEMs with regulated sub-nano ion channels for highly selective anion separation and enhanced acid recovery.^25^ In summary, densifying the membrane proves to be effective in reducing water uptake and consequently mitigating H^+^ leakage.

Building on previous research, this study aims to design in situ crosslinked AEMs that can increase the membranes’ ion exchange capacity (IEC) and simultaneously improve their density for acid blocking. To achieve this, 4-chloromethylstyrene (VBC) was introduced as a side chain onto a polymer backbone. The carbon–carbon double bonds in the side chains can be easily in situ cross-linked during the high-temperature membrane preparation process, leading to higher membrane density and lower water uptake.^26,27^ The study focused on evaluating how different densities of membranes affect acid-blocking properties in ED processes. The physical and chemical properties of the prepared AEMs were systematically investigated, and acid concentration experiments using ED process were conducted to assess their acid-blocking ability. The high IEC and cross-linking degree of the AEMs are expected to reduce water uptake, enhance Donnan exclusion of H^+^ ions, and facilitate counterion transportation for efficient acid blocking. Additionally, an optimized membrane is selected for scaled-up production and further assessment. The evaluation demonstrates that the upscaled membrane possesses commendable acid-blocking characteristics suitable for widespread implementation.

## Discussion

### Chemical Structure of Polymers

**Figure 1:**
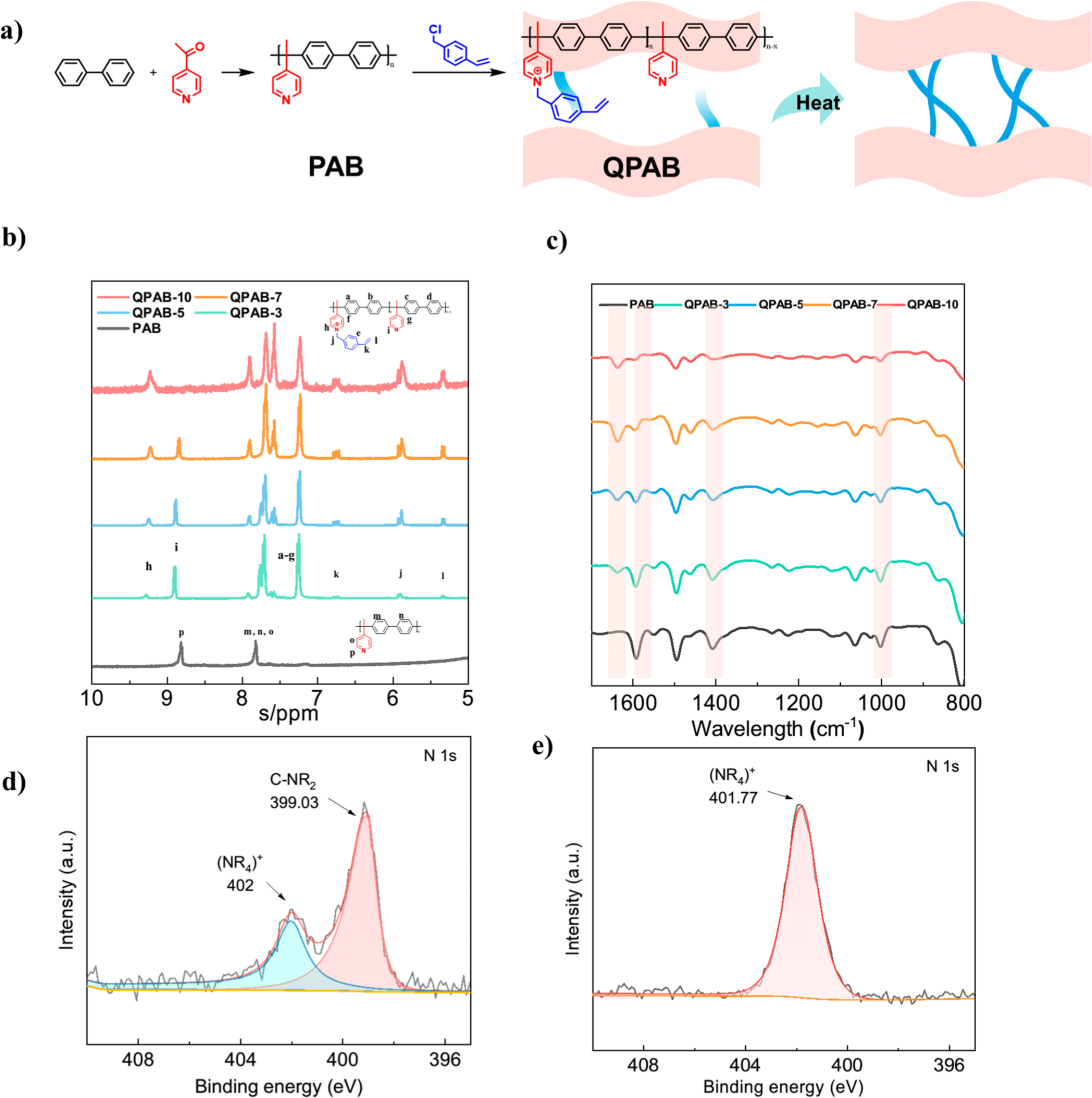
a) The synthesis of the hydrogen-block anionic membrane QPAB-*x* and the in situ crosslinking reaction of the polymer; b) ^1^H NMR spectrum of polymers; c) The ATR-FTIR spectra of polymers; The N 1s XPS spectra of QPAB-3 d) before and e) after immersing in 0.5 mol L^–1^ H_2_SO_4_ solution.

**Figure 2:**
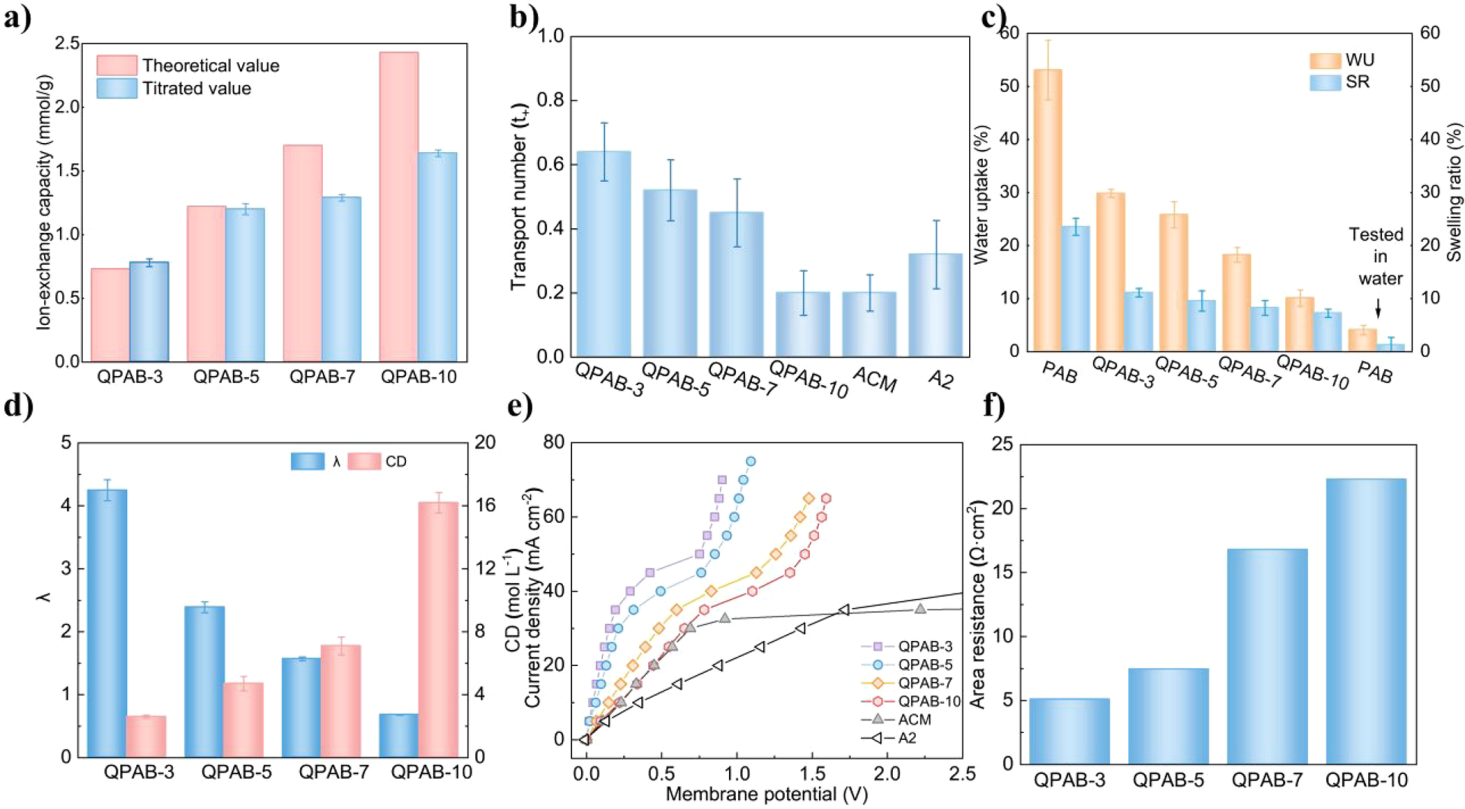
a) The theoretical and titrated IEC of QPAB-*x* membranes; b) The H^+^ transport number of membranes in H_2_SO_4_ tested solution; c) The water uptake and swelling ratio of prepared membranes in 0.5 mol L^–1^ H_2_SO_4_; d) The hydration number (λ) and the fixed charge density (CD) of QPAB-*x* membranes; e) The *I*–*V* curves tested in 0.1 mol L^–1^ H_2_SO_4_; f) The area resistances of QPAB-*x* membranes.

The synthesis procedure for the PAB and QPAB polymers, as well as a visual representation of the in situ crosslinking reaction, are illustrated in Figure 1a. Through a quaternization reaction, the side chain VBC is grafted onto the polymer backbone. During the heating process of membrane formation, the carbon–carbon double bonds at the end of the side chain undergo an in situ cross-linking reaction, tightly linking the backbone and enhancing compactness of QPAB-*x* (*x* = 3, 5, 7, 10, the numbers represent the feeding ratio 30%, 50%, 70% and 100% separately) membranes. The ^1^H NMR spectra of the PAB and QPAB-*x* polymers are shown in Figure 1b. For the PAB polymer, hydrogen near pyridine nitrogen (p position) is responsible for the peak around 8.9 ppm. Hydrogen chemical environments at m, n, and o positions contribute to the peak around 7.8 pm. For QPAB-*x* polymers, the quaternization induces a shift in the position of the pyridine nitrogen from its original location in the PAB polymer, resulting in the emergence of a new peak (h peak) on the spectrum in Figure 2b. Additionally, new peaks appear near the l (5.4 ppm), j (5.9 ppm), and k (6.8 ppm) positions, corresponding to the hydrogens on the side chains. Integration of these peaks at positions l, j, k, allowing for determination of a peak area ratio of 2:2:1, reflecting the hydrogen elemental ratio at these specific positions. This observation confirms successful attachment of VBC side chains onto the PAB backbone through quaternization reaction. Furthermore, integration of the peaks at positions h and i based on their respective areas enables calculation of the actual grafting ratio for VBC. The calculated grafting ratio closely matches the feeding ratio (30%, 50%, 70%, and 100%) used during injection of VBC under prescribed experimental conditions.

The ATR-FTIR spectra of PAB and QPAB-*x* membranes are shown in Figure 1c. The peak at 915 cm^–1^ suggests the presence of C–N bonds formed from quaternary ammonium group production, while the peak at 1640 cm^–1^ indicates the formation of pyridine groups after reaction. Notably, with an increase in VBC content, both peaks at 915 cm^–1^ and 1640 cm^–1^ become more pronounced. Meanwhile, there is a gradual decline in intensity observed for the pyridine peak at 1590 cm^–1^, further confirming successful quaternization.^28^ Additionally, upon heating, the presence of the −CH_2_– group resulted in an absorption band at 1460 cm^–1^. The intensity enhancement of this absorption band is indicative of methylene production through cross-linking reaction occurring at the C=C double bond. Moreover, an increase in VBC content leads to a proportional augmentation of this absorption band, suggesting a higher degree of cross-linking.^29,30^

To investigate how acid environments affect QPAB-*x* polymer properties, XPS characterization was conducted on QPAB-*x* polymers (Figure S1). As shown in Figure 1c, N 1s XPS spectra for QPAB-3 polymer under neutral conditions exhibit two split peaks: one attributed to pyridine group (C-NR_2_) at 399.03 eV and the other to the quaternary ammonium group (NR_4_)^+^ at 402 eV.^31−33^ This result serves as additional evidence supporting successful quaternization of PAB polymer. Furthermore, Figure 1d displays N 1s XPS spectra of QPAB-3 polymer under acidic conditions where only one peak is observed around 401.77 eV corresponding to quaternary ammonium group, indicating that original pyridine groups were protonated upon immersion in sulfuric acid solution,^34^ leading to disappearance of pyridine group peaks.

### Basic Membrane Properties

The comparison depicted in Figure 2a demonstrates a direct correlation between increased VBC content and elevated quaternary ammonium group content, leading to enhanced IEC levels for QPAB-*x* membranes.^19^ However, it was observed that the calculated IEC from ^1^H NMR spectra exceeded its actual measured value. This observation suggests that certain functional groups within these membranes exhibit limited participation in ion exchange process due to their tightly crosslinked structure, impeding efficient exchanging of specific functional groups between Cl^–^ and SO_4_^2–^ ions. Consequently, some Cl^–^ ions remain unexchanged and undetectable. The open circuit voltage can be obtained by fitting the measured current-voltage curves as shown in Figure S2, thus the ion transport number can be calculated. The H^+^ transport number of QPAB-*x* membranes, as well as two commercial membranes A2 and ACM, is shown in Figure 2b. As the side chain content increases in QPAB-*x* membranes, their ability to facilitate H^+^ ions transport gradually declines, indicating a reduction in their H^+^ ions transport numbers. This suggests that increasing membrane densities by enhancing carbon–carbon double bond content within side chains effectively reduces H^+^ ions transport number under acidic conditions while improving acid-blocking capabilities.

The WU and SR of the PAB and QPAB-*x* membranes were examined after immersing them in a 0.5 mol L^–1^ H_2_SO_4_ solution for 24 h, as shown in Figure 2c. In acidic environments, higher IEC resulted in increased WU and SR of QPAB-*x* membranes, with the QPAB-10 membrane showing the lowest values. Generally, greater IEC led to increased WU and SR of the membrane. However, it is worth noting that the presence of carbon–carbon double bonds in the side chain of QPAB-*x* membranes caused enhanced cross-linking degree while increasing IEC, resulting in densification of the membrane structure and inhibition of its swelling ability. Moreover, re-protonation of pyridine groups within QPAB-*x* membranes when immersed in sulfuric acid also affected their WU and SR. Increasing VBC content led to more quaternary ammonium group sites but fewer tertiary amine group sites due to protonation changes. Consequently, all tertiary amine group sites were protonated after immersion in sulfuric acid, leading to a comparable number of hydrophilic groups among different QPAB-*x* membranes. To demonstrate the protonation of tertiary amine sites in acid solutions, enhancing membrane hydrophilicity, the PAB membranes were immersed separately in water and a 0.5 mol L^–1^ H_2_SO_4_ solution to test their WU and SR. The results showed minimal swelling of the PAB membrane without ion exchange groups in water, but significant increases in both WU and SR after immersion in an acidic environment, indicating that QPAB-*x* membranes with tertiary amine sites will experience increased WU and SR under acidic conditions.

The hydration number (λ) represents the average water molecule bonding to each charged functional group in the ion exchange membrane, indicating its water-absorbing capacity. As shown in Figure 2d, QPAB-3, QPAB-5, QPAB-7 and QPAB-10 membranes have λ values of 4.25, 2.39, 1.57, and 0.68 respectively. Interestingly, as the membrane density increases, there is a significant reduction in λ for QPAB-*x* membranes due to enhanced cross-linking that reduces water molecules near ion-exchange sites within these membranes. The fixed charge density (CD) represents the average number of ion-exchange groups per unit of bound water after moisture absorption by the membrane material; this value affects both Donnan exclusion effect and counterion transport rate across the membrane. Notably among all the QPAB-*x* membranes, the QPAB-10 membrane exhibits a higher CD value of 16.2 which enhances anion transport capability while also exerting a stronger Donnan exclusion effect on H^+^ ions.

Typically, *I*–*V* curves consist of three regions: the ohmic region, the limiting current region, and the over-limiting region.^35^ In the ohmic region, current density is directly proportional to voltage. Once reaching the limiting current density (LCD), concentration polarization occurs due to ion depletion at the membrane-solution interface. This leads to a sharp increase in transmembrane voltage, stabilizing the *I*–*V* curves and forming a plateau region.^36−39^ Therefore, it is crucial not to exceed LCD when applying current density in ED processes. In a 0.1 mol L^–1^ sulfuric acid solution, clear ohmic and plateau regions are observed (Figure 2e). QPAB-*x* membranes exhibit LCDs ranging from 30 mA cm^–2^ to 50 mA cm^–2^, both higher than those of ACM and A2 membranes. The *I*–*V* curves reveal that as IEC increases so does LCD for QPAB-*x* membranes. The magnitude of LCD also depends on solution concentration; higher concentrations generally result in an increased LCD.^40−42^ The LCD tested in 0.2 mol L^–1^ sulfuric acid solution (Figure S3) is obviously higher than that of 0.1 mol L^–1^ sulfuric acid solution. Furthermore, area resistance was calculated based on *I*–*V* curves obtained in a 0.1 mol L^–1^ sulfuric acid system (Figure 2f). Among all tested QPAB-*x* membranes, the QPAB-10 membrane exhibits relatively higher area resistance but still falls within an acceptable range. In addition, the *I*–*V* curves of the sodium chloride system were tested (Figure S4), the difference from the results tested in sulfuric acid system is that the higher the IEC, the lower the resistance of the QPAB-*x* membranes, which can be attributed to the properties of QPAB-*x* as anion exchange membranes.

The mechanical properties of QPAB-*x* membranes were also tested. The stress-strain curves showed that QPAB-10 had good mechanical strength in water and acid environments (Figure S5), which is because the crosslinking of the side chain strengthens the bonding between the backbones, resulting in enhanced resistance to membrane breakage.

### Electrodialysis

**Figure 3:**
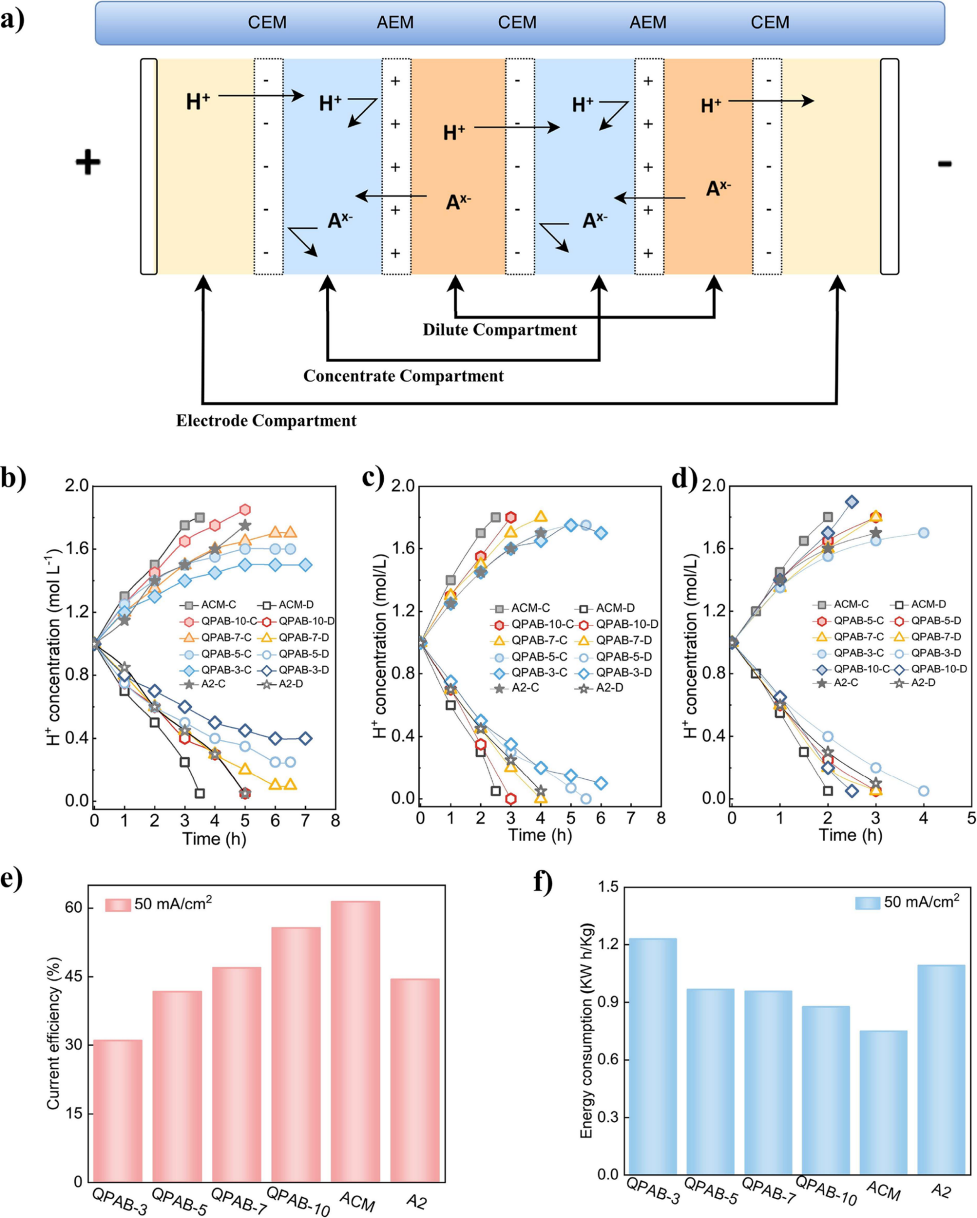
a) The structure of ED stack for acid concentration; The H^+^ concentration variation curve of concentrated chamber and dilute chamber in current density of b) 30 mA cm^–2^; c) 40 mA cm^–2^; d) 50 mA cm^–2^; e) The current efficiency of ED in 50 mA cm^–2^ current density; f) The energy consumption of ED in 50 mA cm^–2^ current density.

The acid-blocking performance of QPAB-*x* membranes was evaluated at three different current densities using ED process as shown in Figure 3a. The results revealed an increase in H^+^ ion concentration in the concentrated chamber and a decrease in the dilute chamber. When the ion concentration of the dilute chamber is reduced to a certain level, the ED stack voltage will increase significantly as shown in Figure S6. Initially, there was a linear rise in H^+^ ion concentration in the concentrated chamber until it reached a plateau due to significant transport of H^+^ ion from the dilute chamber. This resulted in minimal differences between both chambers’ H^+^ ion concentrations, facilitating easy permeation of H^+^ ions through the cation exchange membrane into the concentrated chamber. As ED operation progressed, fewer ions were transported from the dilute chamber, leading to a slower rate of ion concentration increase in the concentrated chamber. Simultaneously, there was an increasing concentration difference between chambers, resulting in the diffusion of H^+^ ions from the concentrated chamber to the dilute chamber. The challenge posed by blocking acid through potential and concentration differences provided by the anion exchange membrane became progressively more difficult, causing some leakage of H^+^ ions into the dilute chamber, particularly under low current density conditions, which reduced efficiency of H^+^ ion concentration and caused leakage into the concentrated chamber. As shown in Figure 3b, QPAB-3, QPAB-5, and QPAB-7 membranes achieve equilibrium at lower current densities while maintaining stable H^+^ leakage and concentration in the concentrated chamber. The leakage of hydrogen ions is slowed down by increasing the current density as shown in Figure 3c. As demonstrated in Figure 3d, in contrast, QPAB-*x*, A2, and ACM membranes reach the concentration end point through H^+^ ion transfer from the dilute chamber. Among these membranes, the QPAB-10 membrane exhibited superior acid-blocking properties compared to others in the QPAB-*x* series, similar to ACM membrane but better than A2 membrane. Increasing IEC by enhancing VBC content and cross-linking degree effectively improved acid-blocking performance. Although the hydrogen ion concentration in the concentrated chamber did not reach the desired 2 mol L^–1^, which can be attributed to water molecule migration during ion transport across the membrane (Figure S7). Notably, the QPAB-10 membrane displayed reduced water migration resulting in higher H^+^ ion concentrations at the electrodialysis end point, particularly at a current density of 50 mA cm^–2^. The efficiency of acid concentration through electrodialysis is primarily influenced by current density: longer times are required at low current densities to reach the ED end point, as shown in Figure 3b-d. For example, for the QPAB-10 membrane, the time taken to reach the concentration end point increased from 2.5 h (50 mA cm^–2^) to 3 h (40 mA cm^–2^) and 5 h (30 mA cm^–2^). Insufficient acid-blocking properties in membranes at low current densities led to an equilibrium of H^+^ ion leakage and left some ions in the dilute chamber. This limitation was evident in QPAB-3, QPAB-5, and QPAB-7 membranes highlighting their restricted acid-blocking capacity.

The current efficiencies and energy consumption were calculated at three different current densities using equation 8, as depicted in Figure 3e and f. The results consistently indicated that the current efficiencies increased sequentially from QPAB-3 to QPAB-10, corresponding to enhanced acid-blocking performance as shown in Figure 3e. Additionally, it was observed that higher current densities led to higher current efficiency for a specific membrane (Figure S8). This can be attributed to the fact that at lower current densities, the H^+^ ion leakage efficiency becomes equal or even surpasses the concentration efficiency when the concentrated chamber reaches a certain level of concentration. A low current efficiency signifies higher energy consumption per unit of time.^18,43,44^ Membranes with inadequate acid-blocking performance necessitate longer operation times, leading to elevated energy consumption levels,^45^ whereas membranes with superior acid-blocking performance exhibit reduced energy consumption levels, as depicted in Figure 3f. The proposed scheme effectively achieves excellent acid-blocking ability while enhancing acid concentration through the ED process. Notably, the QPAB-10 membrane demonstrates high current efficiencies and low energy consumption, outperforming commercial A2 membrane.

### Electrodialysis in Hydrochloric Acid System

**Figure 4:**
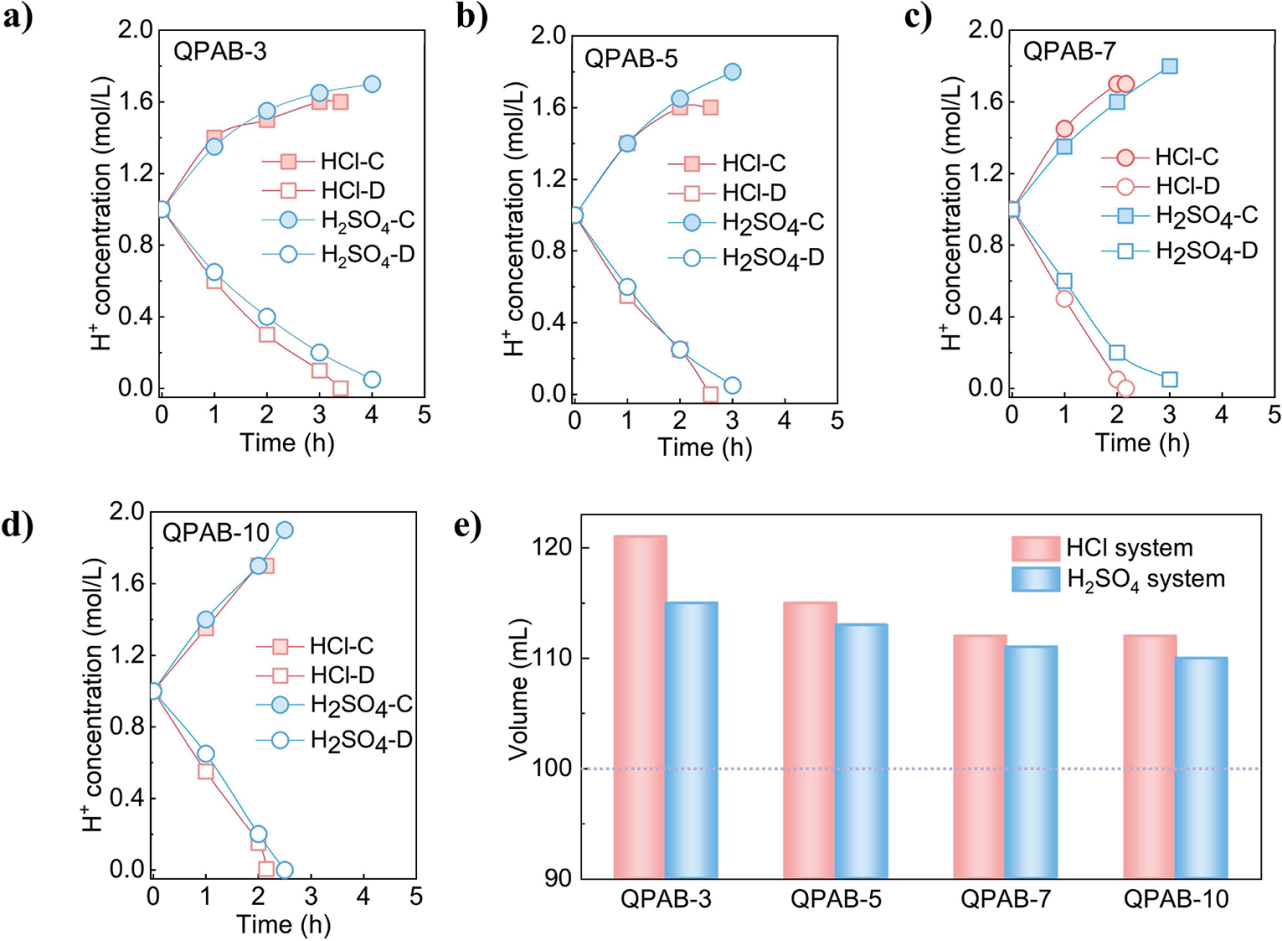
a) QPAB-3; b) QPAB-5; c) QPAB-7 and d) QPAB-10 acid-blocking capacity comparison of the H_2_SO_4_ system and HCl system; e) The water transfer of the concentrated chamber in two systems.

To investigate the effects of different acids on the acid-blocking performance of QPAB-*x* membranes, the concentrated chamber was altered by substituting a 0.5 mol L^–1^ sulfuric acid solution with a 1 mol L^–1^ hydrochloric acid solution, changing the anionic species. ED experiments with hydrochloric acid concentration were conducted under identical conditions, and the results are depicted in Figure 4a-d. the QPAB-*x* membrane displayed similar acid-blocking properties in both hydrochloric acid and sulfuric acid systems. Nonetheless, the hydrochloric acid system achieved the concentration end point more quickly due to the smaller hydration radius of Cl^-^ ions, facilitating their passage through QPAB-*x* membranes compared to SO_4_^2–^ ions. To maintain electroneutrality, H^+^ ions were encouraged to transport across membrane to some extent in the hydrochloric acid system, slightly accelerating their concentration rate. Furthermore, at the concentration end point, the H^+^ ion concentration in concentrated chamber was lower in the hydrochloric acid system than in the sulfuric acid system due to increased water migration resulting from greater ion movement within it, as demonstrated in Figure 4e.^46−48^

The current efficiency and energy consumption of hydrochloric acid system were also calculated as shown in Figure S9, which showed the same regularity as the result of sulfuric acid system.

### Scale-up Membrane and Application

**Figure 5:**
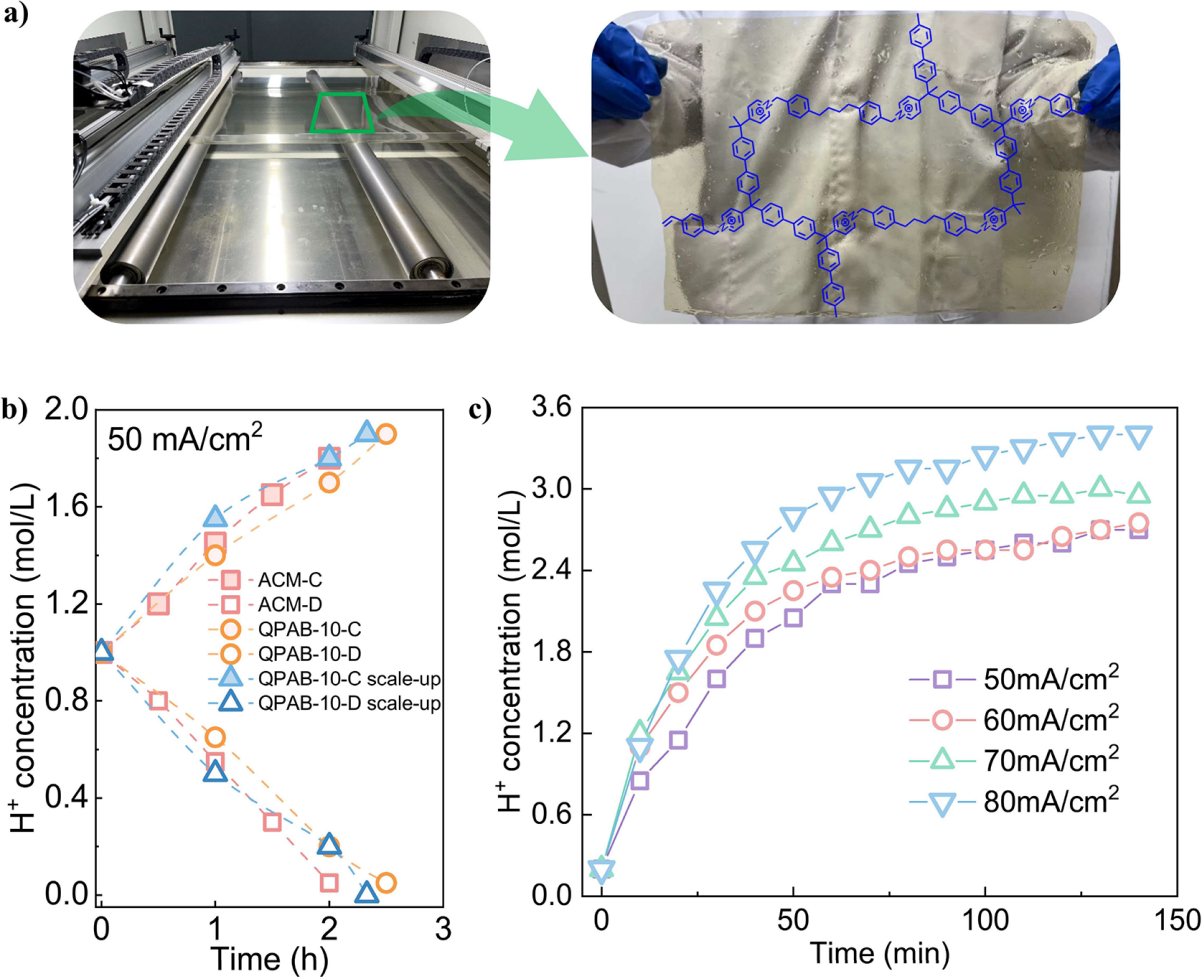
a) The preparation process of scale-up membrane; b) The comparison acid-blocking capacity of ACM and QPAB-10; c) The result of limiting acid concentration experiment.

Considering area resistance, limiting current density and acid-blocking capacity, the QPAB-10 membrane was selected for large-scale production, and the process is shown in Figure S10. A 25 cm × 20 cm QPAB-10 membrane was successfully prepared (Figure 5a) and evaluated for reproducibility through ED acid concentration experiments at the laboratory level. The successful synthesis of the scaled-up QPAB-10 membrane is confirmed by ^1^H NNR result (Figure S11), while the tensile experiments futher demonstrate its superior mechanical properties (Figure S12). The ED results demonstrated that the scaled-up membrane maintained excellent acid-blocking ability and reproducibility (Figure 5b). Furthermore, the industrial potential of the scaled-up membrane was evaluated through conducting limiting acid concentration experiments using a larger ED membrane stack which has 10 repeat units (the effective area is 189 cm^2^). The detailed experimental parameters are shown in Figure S13. Results depicted in Figure 5c indicate that an increase in current density positively influences enhancing limiting acid concentration. At the current density of 80 mA cm^–2^, a final concentration of 1.75 mol L^–1^ for sulfuric acid with an initial concentration of 0.1 mol L^–1^ was achieved, showcasing promising application potential. More test results of the amplified membrane are shown in Figure S14 and Figure S15, which also showed the QPAB-10 membrane’s excellent acid blocking properties and is superior to commercial membrane A2.

## Conclusion

The QPAB-*x* series of in situ crosslinked AEMs were synthesized through a quaternization reaction. The results indicated that higher crosslinking degree in the QPAB-*x* membranes led to lower hydration numbers and increased fixed charge densities. On the one hand, reducing the water content limited the transport of H^+^, while on the other hand, the higher fixed charge density enhanced the Donnan exclusion effect on H^+^. Consequently, among all prepared membranes, the QPAB-10 membrane with the highest crosslinking degree and IEC exhibited the lowest H^+^ transport number. Compared to commercial ACM and A2 membranes, the QPAB-10 membrane demonstrated superior acid resistance with higher limiting current density and lower area resistance. The QPAB-10 membrane displayed excellent acid blocking property along with improved current efficiency and reduced energy consumption compared to other QPAB-*x* membranes; its performance was compared to commercial ACM and outperformed commercial membrane A2. Furthermore, when scaled up for practical applications, the QPAB-10 membrane exhibited commendable acid blocking capacity while maintaining favorable mechanical characteristics.

## Experimental Section

### Materials

Biphenyl (98%), 4-acetylpyridine (98%), 4-vinylbenzyl chloride (VBC, 90%), trifluoromethanesulfonic acid (TFSA, 97%), trifluoroacetic acid (TFA, 98%), *N*-methyl-2-pyrrolidone (NMP, 98%) and dichloromethane (DCM, 99%) were purchased from Energy Chemistry Co. Ltd and used without further purification. Sodium chloride (NaCl, 99%), sodium sulfate (Na_2_SO_4_, 99.0%) sulfuric acid (H_2_SO_4_, 95.0% ∼ 98.0%), hydrochloric acid (HCl, 36.0%∼38.0%), and sodium hydroxide (NaOH, 99.5%) were purchased from Sinopharm Group Chemical Reagent Co. Ltd. Two commercial membranes, ACM (acid-blocking anion exchange membrane) and CM-2 (cation exchange membrane), were obtained from Astom Crop Company, Japan.

### Synthesis of Poly(alkyl-biphenylpyridine)

The poly(alkyl-biphenylpyridine) (PAB) was synthesized via polyhydroxy alkylation.^49,50^ Initially, biphenyl (7.71 g) was dissolved in dichloromethane (12.5 mL). Then, 4-acetylpyridine (7.87 g) was added and thoroughly stirred. The reaction system was cooled to 0 °C using an ice-water bath and a recirculating condenser. While stirring constantly, TFSA (60 mL) and TFA (2.75 mL) were gradually added dropwise, maintaining the temperature near 0 °C. After an 8-hour reaction period, the resulting viscous system was poured into excess water to yield a yellow fibrous product. The product was repeatedly washed with deionized water and dried in a vacuum oven at 60 °C. Subsequently, the resulting polymer was immersed in a 1 mol L^–1^ NaOH solution for 48 h. Then the polymer was washed with a substantial amount of deionized water until neutral. Finally, the polymer was dried in an oven at 60 °C for 24 h to obtain the desired PAB polymer.

### Synthesis of Quaternized Poly(alkyl-biphenylpyridinine)

The four 100 mL round-bottomed flasks were thoroughly cleaned and dried. Each flask was then filled with 4 g of PAB and 36 g of NMP. After the polymers had dissolved completely, VBC was added to each flask in specific quantities: 0.71, 1.18, 1.66, and 2.36 g, respectively. The flasks were subsequently placed in a thermostatic water bath set at a temperature set to 30 °C and stirred continuously for 48 h, resulting in the successful formation of quaternized PAB solutions (QPAB-*x*), named as QPAB-3, QPAB-5, QPAB-7, and QPAB-10. The QPAB-*x* solution was applied onto a pristine glass plate, which was then heated on a heating table at a temperature of 80 °C for several hours to facilitate solvent evaporation and formation of the QPAB-*x* series membranes. Subsequently, the glass plate was submerged in water for 4 h to easily remove the membranes from it.

### ^1^H Nuclear Magnetic Resonance Spectra (^1^H NMR), Attenuated Total Reflection-Fourier Transform Infrared Spectroscopy (ATR-FTIR) and X-ray Photoelectron Spectroscopy (XPS)

The chemical structures of the prepared polymers were characterized using ^1^H NMR and ATR-FTIR spectra. The ^1^H NMR spectra were recorded using a Bruker Avance 400S spectrometer, with dimethyl sulfoxide-d6 as solvent and tetramethylsilane as the standard. The ATR-FTIR spectra were recorded by Nicolet iS 10, Thermo Fisher Scientific. The XPS was tested by Kratos Axis supra+, the X-ray excitation source was a monochromatic Al Ka (hυ =1486.6 eV) with a test power of 150 W and an x-ray beam spot of 300 μm × 700 μm.

### Ion Exchange Capacity

The membrane samples were initially immersed in a 0.5 mol L^–1^ NaCl solution for 24 h to ensure complete adsorption of Cl^-^ ions by the quaternary ammonium groups present in membranes. The samples were then washed thoroughly with deionized water to remove any residual Cl^-^ ions. By subjecting the dried membranes to a temperature of 80 °C for 8 h, the weight of the membranes in their dried state (W_dry_, g) was measured and recorded. Subsequently, the dried membranes were immersed in a 0.5 mol L^–1^ Na_2_SO_4_ solution for 24 h, thus facilitating the complete exchange of Cl^–^ ions within the membranes with SO_4_^2–^ ions present in the solution.^51^ The quantification of Cl^-^ ions within the solution was achieved by means of titration, utilizing a potentiometric titrator and a standard 0.01 mol L^–1^ AgNO_3_ solution. The volume of the AgNO_3_ solution consumed during the titration process was also documented. Finally, the IEC of the membranes can be calculated through the utilization of equation 1:
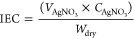
1

### Water Uptake, Swelling Ratio

The water uptake (WU) and swelling ratio (SR) can be quantified concurrently. The membrane samples were immersed in 0.5 mol L^–1^ H_2_SO_4_ for 24 h to ensure complete saturation. After removing the surface solution, the length (*L*_wet_) and weight (*W*_wet_) of the wet samples were measured. The samples were then dried at 80 °C for 8 h, and the length (*L*_dry_) and weight (*W*_dry_) of the dried samples were measured. The WU and SR of the samples can be calculated using equations 2 and 3, respectively.
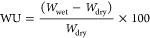
2
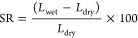
3

### Current–Voltage Curves and Membrane Area Resistance (*R*_m_)

The current–voltage (*I*–*V*) curves were obtained using a four-chamber apparatus.^52^ The *I*–*V* curves were measured in sodium chloride and sulfuric acid solutions. The apparatus consisted of two side chambers filled with a sodium sulfate solution (0.3 mol L^–1^) and two middle chambers filled with either sodium chloride solution (0.2 mol L^–1^) or sulfuric acid solution (0.2 mol L^–1^), circulating through a peristaltic pump. A constant potentiometer was employed to supply the platinum electrodes at the ends of the apparatus, providing a gradually increasing current. The voltage change across the investigated membrane was measured by using Ag/AgCl electrodes. The membrane’s area resistance can be determined through the analysis of the ohmic region present in the *I*–*V* curve. The process involves fitting a linear regression to the ohmic region, allowing for the determination of the slope. The reciprocal of this slope yields the precise value of the area resistance.

### Transport Number (*t*_+_)

The transport number was measured using a two-chamber apparatus. The QPAB-*x* membranes were placed in the middle of the chambers, with solutions of H_2_SO_4_ at concentrations of 0.01 and 0.1 mol L^–1^ filling the chambers, respectively. Ag/AgCl electrodes were used to measure the potential on both sides of the membranes. The transport number (*t*_+_) can be calculated using equation 4:
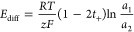
4where *E*_diff_ is the potential of the membrane, *z* is the ionic charge (1 for H^+^), *T* is the absolute temperature, *a*_1_ and *a*_2_ are the activities of the electrolyte in the solutions, *R* is the universal gas constant, and *F* is the Faraday constant.

### Hydration Number (λ)

The hydration number (λ) is defined as the number of water molecules bound per unit of functional group, and the λ is related to the IEC and WU of the membranes, which can be calculated by equation 5:
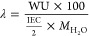
5where *M*_H_2_O_ is the molecular weight of water.

### Fixed Charge Density (CD)

The fixed charge density is defined as a concentration of fixed charge group per unit volume of bound water, which can be calculated by equation 6:
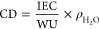
6where ρ_H_2_O_ is the molecular weight of water.

### Electrodialysis

**Scheme 1:**
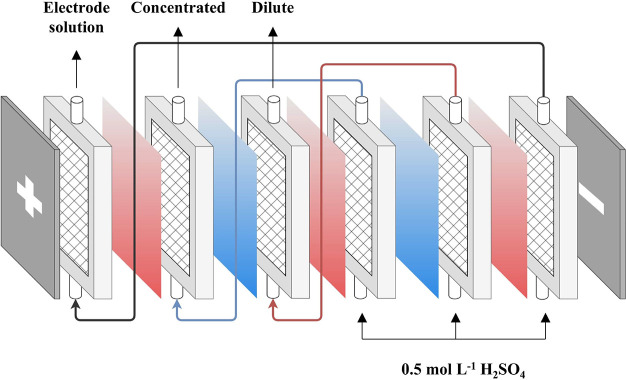
ED Stack of Acid Concentration

The experiments involving dialysis acid concentration were conducted using the apparatus which comprises three CM-2 membranes and two AEM membranes, which were placed alternately to create four compartments. These compartments included two concentrated chambers and two dilute chambers, with cation exchange membranes on both sides and electrode plates forming two electrode chambers. The concentration, dilute, and electrode chambers were interconnected through conduits respectively (Scheme 1).

Each chamber was supplied with 100 mL 0.5 mol L^–1^ sulfuric acid solution, which circulated at a flow rate of 200 mL min^–1^. The membranes had an effective area of 21 cm^2^ and a spacing of 1 cm. To induce ion migration, the electrochemical workstation provided constant currents of 30, 40, and 50 mA cm^–2^. As a result, the voltage increased due to the continuous decrease in hydrogen ions in the dilute chamber. The experiment concluded either when the voltage reached the maximum operating voltage of the power supply (36 V) or when the hydrogen ion concentration in the concentrated chamber remained constant during two consecutive hours of measurement.

Throughout the experiment, the potential between the two meters was recorded hourly. Additionally, a sample of 200 μL was extracted from each concentration and dilute chamber using a pipette gun and transferred to a beaker. Acid–base titration was conducted by adding 0.01 mol L^–1^ NaOH solution from the buret, with the indicator being 1% g mL^–1^ phenolphthalein reagent. The performance of QPAB-*x* membranes in terms of current efficiency (η), energy consumption (*E*), and changes in H^+^ concentration in the concentration and dilute chambers was compared to that of commercial membranes. Current efficiency and energy consumption can be calculated from equation 7 and equation 8, respectively:
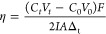
7
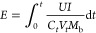
8where *C*_0_ and *C*_*t*_ represent the acid concentration in the concentrated compartment corresponding to initial time and time *t*, respectively; *V*_0_ and *V_t_* represent the concentrated chamber solution volume corresponding to the initial time and time *t*, respectively; *F* is the Faraday constant (96,485 C mol^–1^); *I* is current density; *A* is the effective membrane area and Δ*t* is the time interval. *E* is the energy consumption (kW h kg ^–1^); U is the voltage between two electrode plates; *M*_b_ is the molecular of H_2_SO_4_.^53^

## Supporting Information Available

XPS spectra characterization, ion transport number, *I*–*V* curves, mechanical property test, details of electrodialysis, water transport, current efficiency, process of preparing scaled-up QPAB-10 membrane, ^1^H NMR, tensile curves of scaled-up QPAB-10 membrane, experimental parameters of limiting concentration experiment, ED results of scaled-up QPAB-10, H^+^ concentration and voltage variation (PDF)

## ABBREVIATIONS

AEM: anion exchange membrane
CEM: cation exchange membrane
ED: electrodialysis
LCD: limiting current density
