# Supplementary material for: Side Chain Crosslinked Anion Exchange Membrane for Acid Concentration by Electrodialysis
Source: Chem Bio Eng. 2024 Jul 10;1(7):647–57. doi: 10.1021/cbe.4c00096 (PMC11835270; doi:10.1021/cbe.4c00096)
Supplement: Supplementary file 1 — be4c00096_si_001.pdf [file be4c00096_si_001.pdf]

# Supporting Information

# Side chain crosslinked anion exchange membrane for acid concentration by electrodialysis

Haoyang He[a, b§], Qian Chen[a§], Rongqiang Fu[c], Zhaoming Liu[c], Liang Ge[a, b*] and Tongwen Xu[a*]

[a] Key Laboratory of Precision and Intelligent Chemistry, School of Chemistry and Materials Science, University of Science and Technology of China, Hefei, 230026, China.

[b] Applied Engineering Technology Research Center for Functional Membranes, Institute of Advanced Technology, University of Science and Technology of China, Hefei, 230088, China.

[c] Shandong Tianwei Membrane Technology Co., LTD, Weifang, 262737, China.

[*] E-mail: geliang@ustc.edu.cn, twxu@ustc.edu.cn

[§]Contributed equally to this work.

## 1. XPS spectra characterization

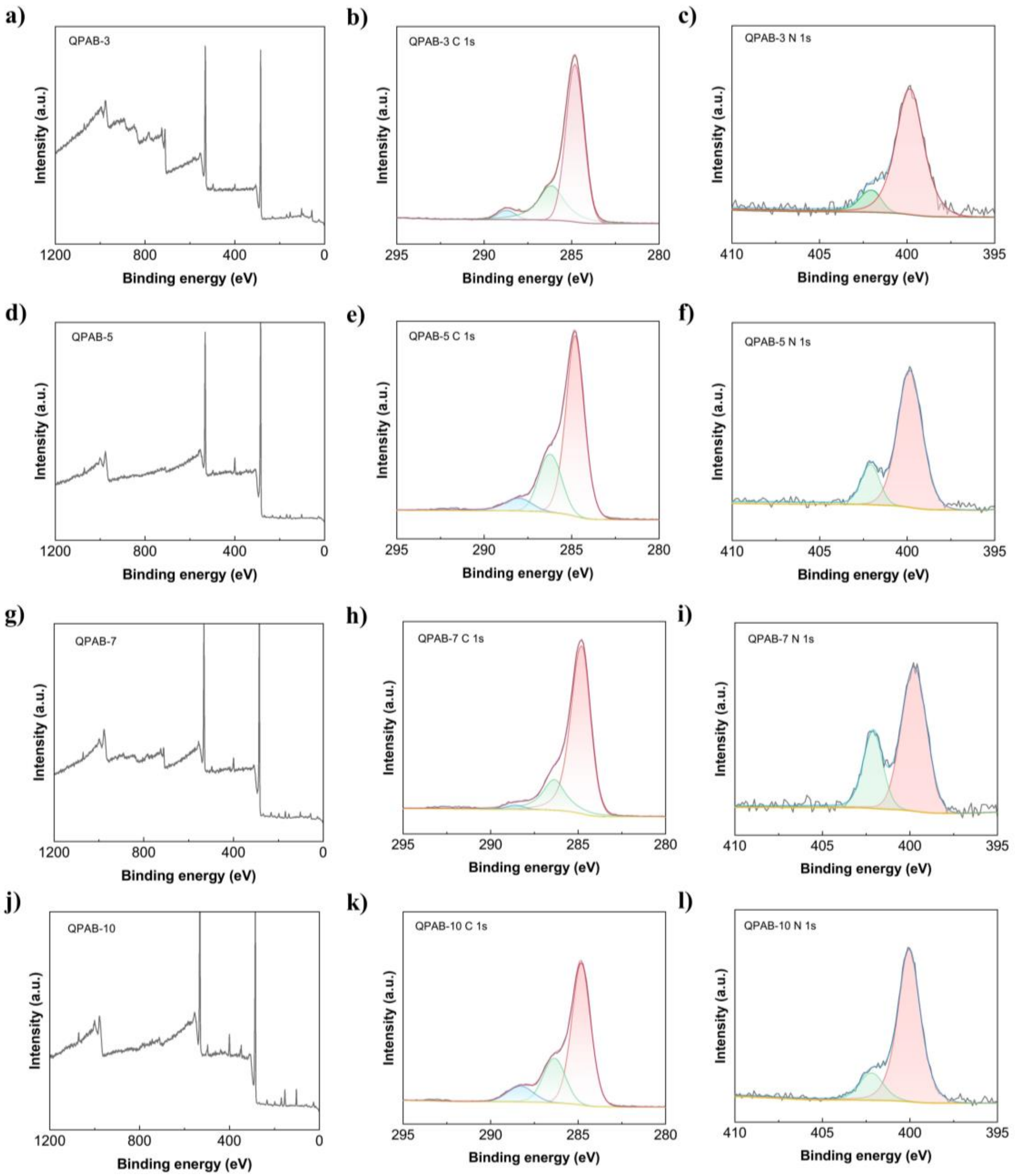


**Figure S1.** a) The a) XPS spectra, b) C 1s XPS spectra and c) N 1s XPS spectra of QPAB-3; The d) XPS spectra, e) C 1s XPS spectra and f) N 1s XPS spectra of QPAB-5; The g) XPS spectra, h) C 1s XPS spectra and i) N 1s XPS spectra of QPAB-7; The j) XPS spectra, k) C 1s XPS spectra and l) N 1s XPS spectra of QPAB-10.

## 2. Ion transport number

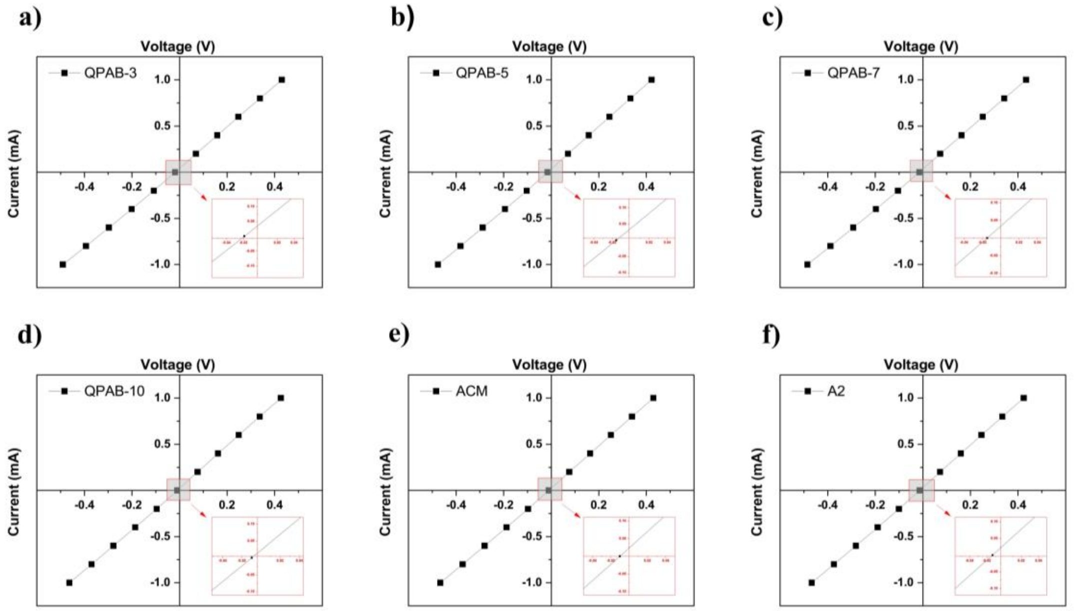


**Figure S2.** The $H^+$ transport number of a) QPAB-3; b) QPAB-5; c) QPAB-7; d) QPAB-10; e) ACM; f) A2.

## 3. I-V curves

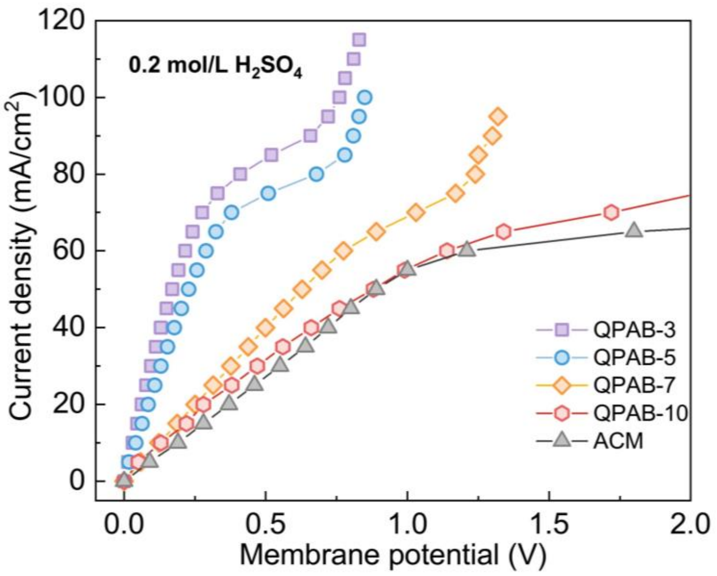


**Figure S3.** The I-V curves tested in 0.2 mol $L^{-1}$ $H_2SO_4$.

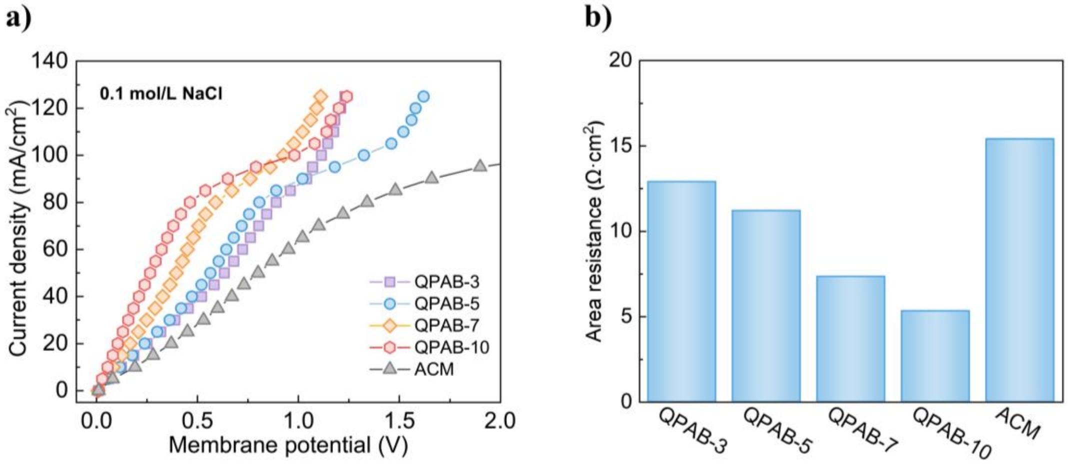


**Figure S4.** a) The I-V curves tested in 0.1 mol $L^{-1}$ NaCl; b) The area resistances of membranes.

## 4. Mechanical property test

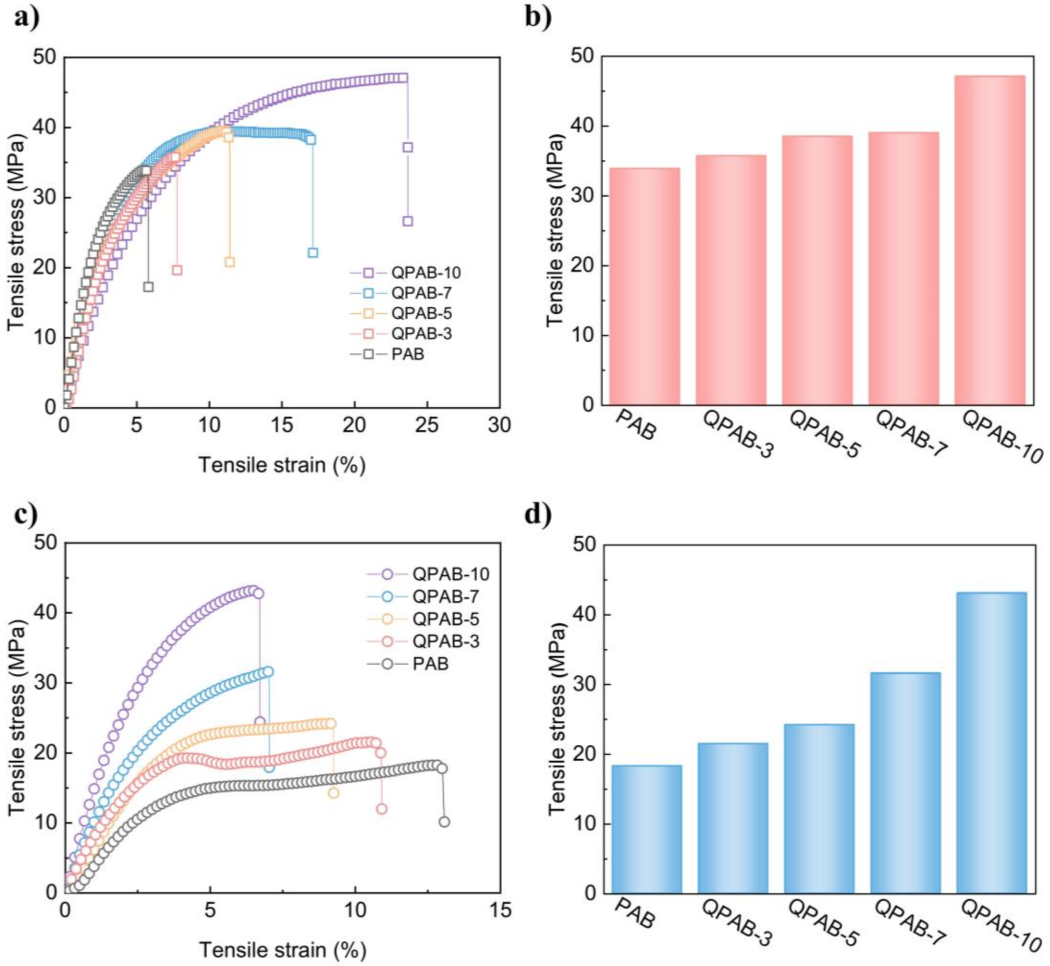


**Figure S5.** The a) tensile curves and b) tensile break stress of PAB and QPAB-x membranes after immersing in water for 24 hours; The c) tensile curves and d) tensile break stress of PAB and QPAB-x membranes after immersing in 0.5 mol $L^{-1}$ $H_2SO_4$ solution for 24 hours.

## 4. Details of electrodialysis

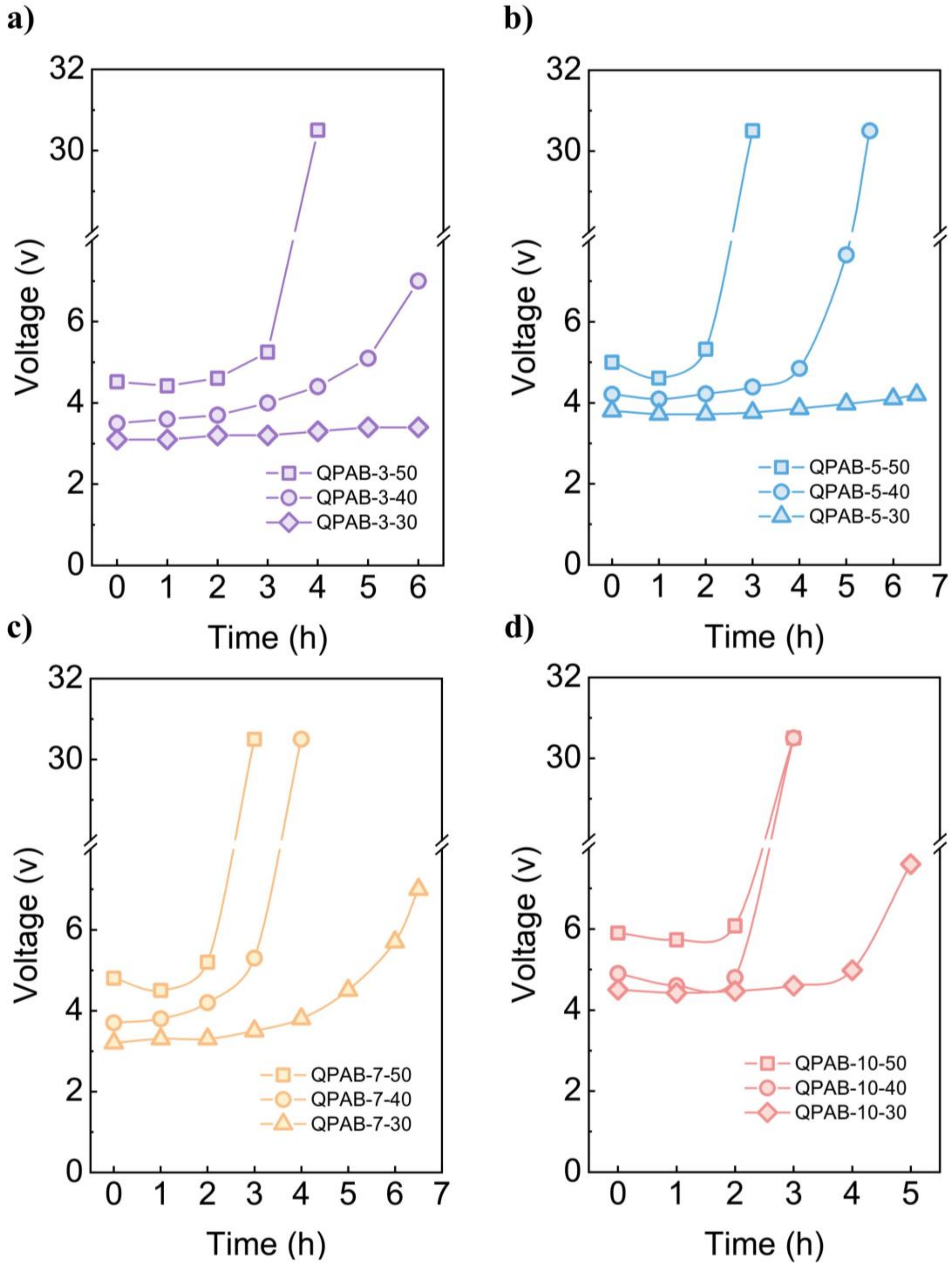


**Figure S6.** The voltage variation curves of a) QPAB-3; b) QPAB-5; c) QPAB-7 and d) QPAB-10 in ED processes.

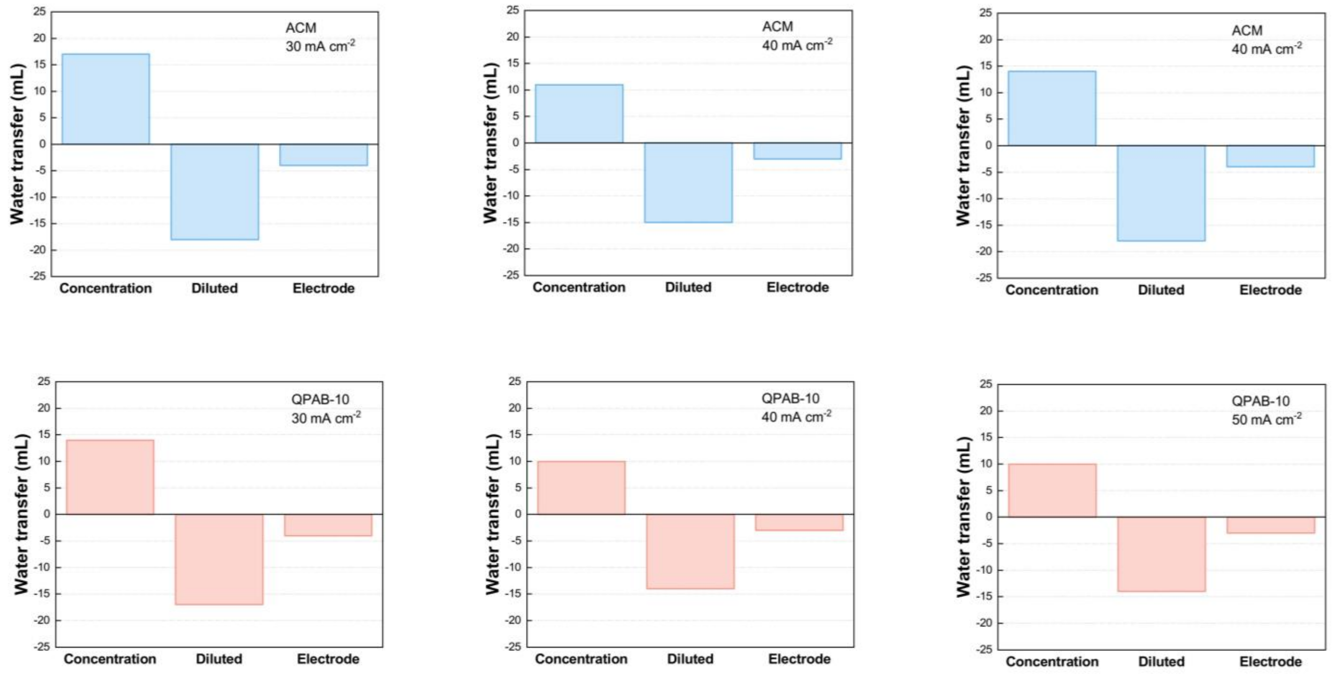


**Figure S7.** The water transport of ACM and QPAB-10 during ED processes.

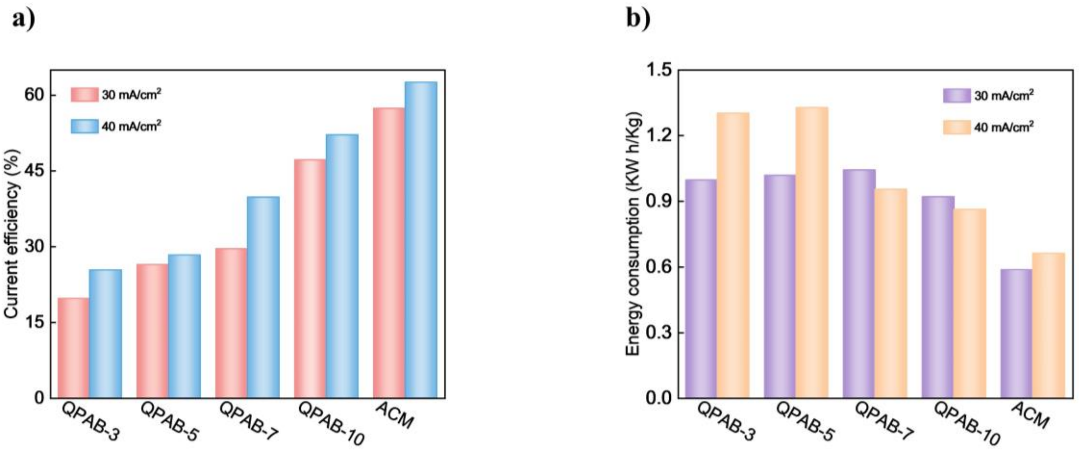


**Figure S8.** a) The current efficiency of ED stack in 30 mA cm$^{-2}$ and 40 mA cm$^{-2}$ current density; b) The energy consumption of ED stack in 30 mA cm$^{-2}$ and 40 mA cm$^{-2}$ current density.

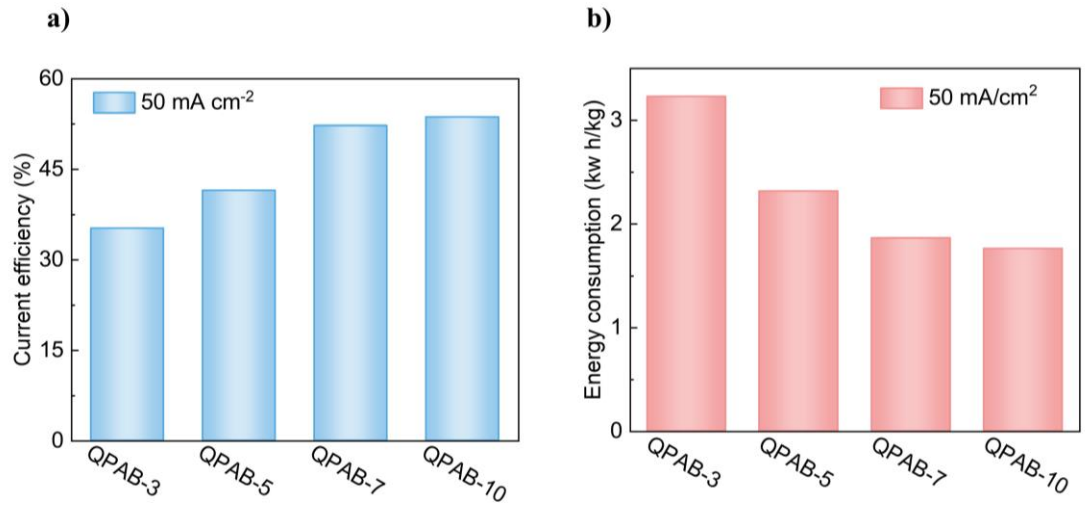


**Figure S9.** a) The current efficiency of ED in HCl system; b) The energy consumption of ED in HCl system.

## 5. Experiment process

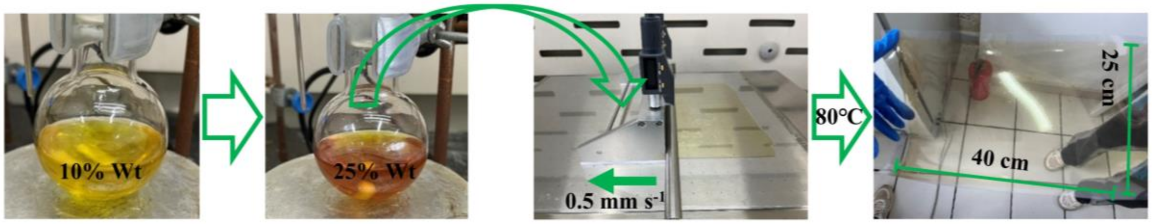


**Figure S10.** The process of preparing scaled-up QPAB-10 membrane.

## 6. $^1$H NMR of scaled-up QPAB-10 polymer

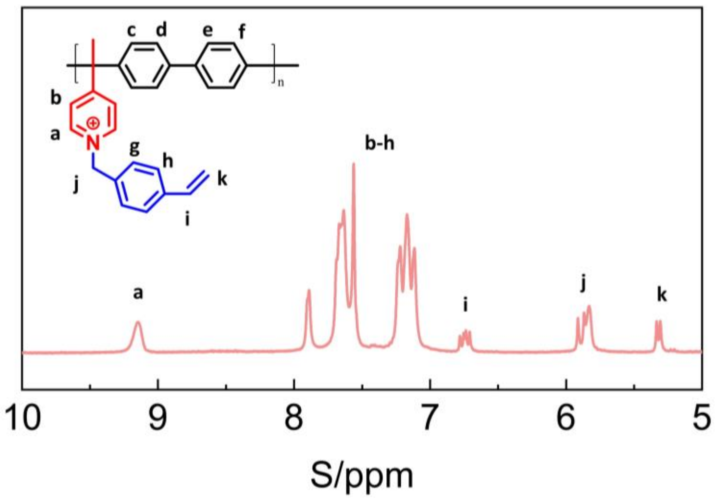


**Figure S11.** The $^1$H NMR of scaled-up QPAB-10 polymer.

## 7. Mechanical property of scaled-up QPAB-10 membrane

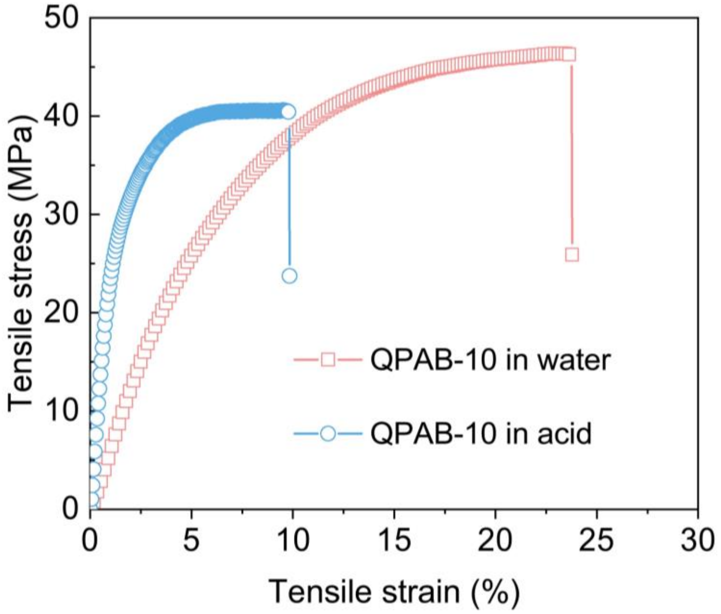


**Figure S12.** The tensile curves of scaled-up QPAB-10 membrane.

## 8. Electrodialysis

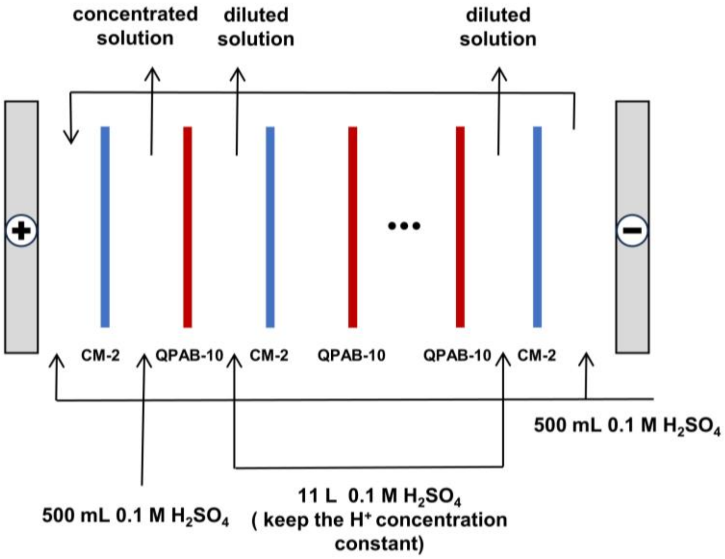


**Figure S13.** The experimental parameters of limiting concentration experiment.

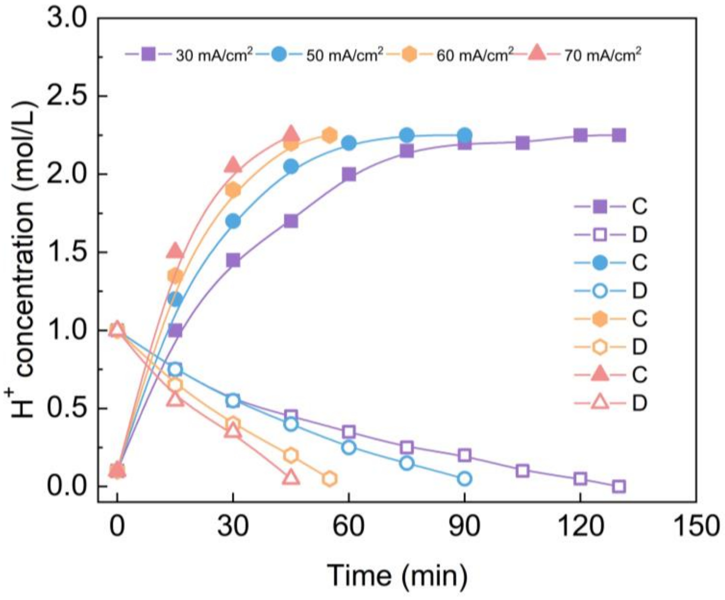


**Figure S14.** The ED results of scaled-up QPAB-10 under the condition of 500 mL 0.5 mol $L^{-1}$ $H_2SO_4$ in concentrated chamber, 2000 mL 0.5 mol $L^{-1}$ $H_2SO_4$ in dilute chamber and 500 mL 0.5 mol $L^{-1}$ $H_2SO_4$ in electrode chamber.

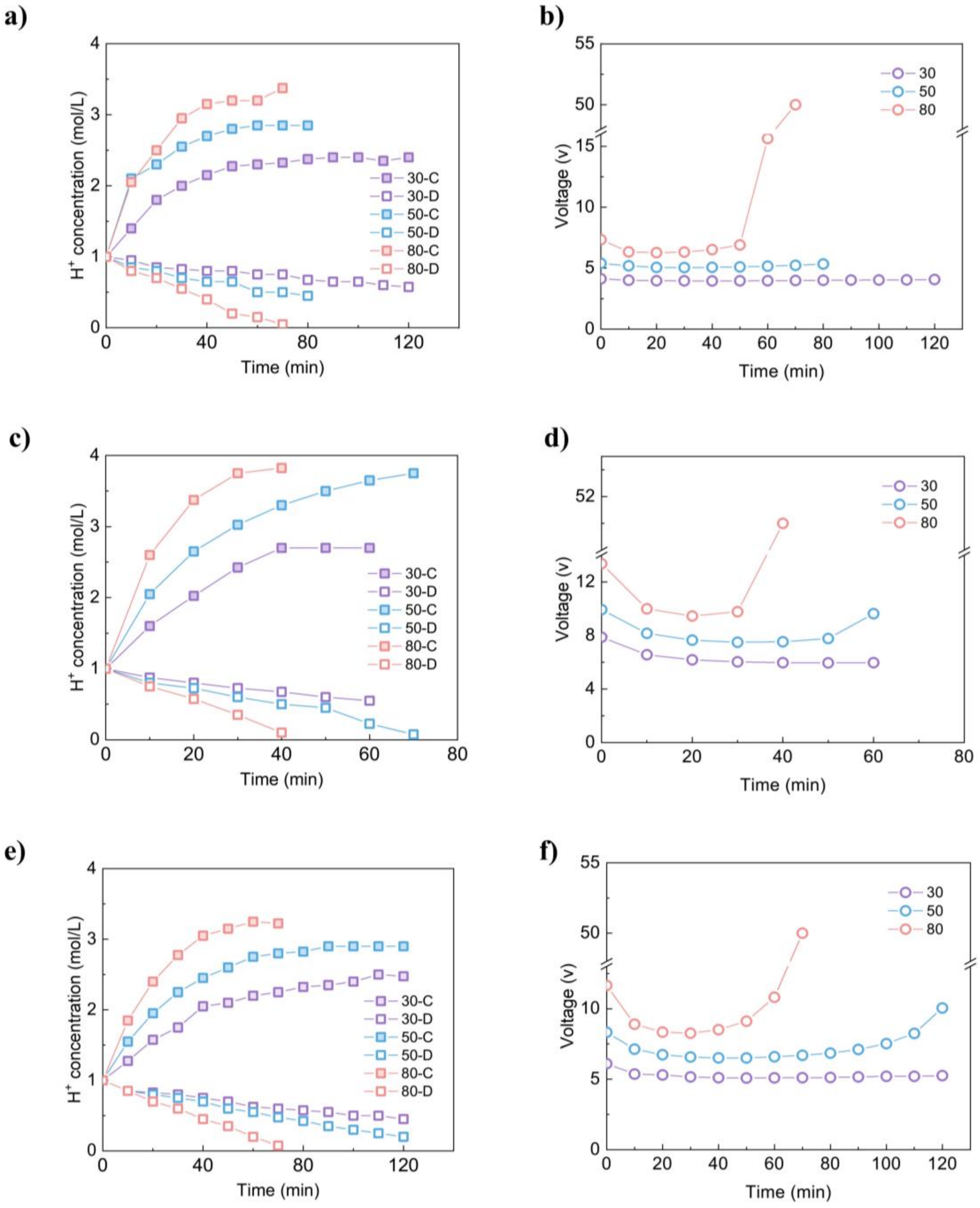


**Figure S15.** The a) $H^+$ concentration and b) voltage variation in ED process using scaled-up QPAB-10; The c) $H^+$ concentration and d) voltage variation in ED process using ACM; The e) $H^+$ concentration and f) voltage variation in ED process using A2. Under the condition of 250 mL 0.5 mol $L^{-1}$ $H_2SO_4$ in concentrated chamber, 2000 mL 0.5 mol $L^{-1}$ $H_2SO_4$ in dilute chamber and 250 mL 0.5 mol $L^{-1}$ $H_2SO_4$ in electrode chamber.
